# Association Between Progranulin (PGRN) Levels in Serum and Cerebrospinal Fluid with Integrated Clinical Indices in Patients with Idiopathic Normal Pressure Hydrocephalus

**DOI:** 10.3390/jcm15156129

**Published:** 2026-08-06

**Authors:** Łukasz A. Poniatowski, Kristin Eske-Pogodda, Agnieszka Siwińska, Mieszko Olczak, Kristian Meinck, Michael J. Fritsch

**Affiliations:** 1 Department of Neurosurgery, Dietrich-Bonhoeffer-Klinikum, Salvador-Allende-Straße 30, 17036 Neubrandenburg, Germany; fritschm@dbknb.de; 2 Institute of Laboratory Diagnostics, Microbiology and Transfusion Medicine, Dietrich-Bonhoeffer-Klinikum, Salvador-Allende-Straße 30, 17036 Neubrandenburg, Germany; eskepk@dbknb.de (K.E.-P.); meinckk@dbknb.de (K.M.); 3 Department of Forensic Medicine, Center for Biostructure Research, Medical University of Warsaw, Oczki 1, 02-007 Warsaw, Poland; agasiwa@gmail.com; 4 Department of Forensic Medicine, Institute of Medical Sciences, Faculty of Medicine, Collegium Medicum, Cardinal Stefan Wyszyński University, Kazimierza Wóycickiego 1/3, 01-938 Warsaw, Poland; mieszkothe1st@hotmail.com

**Keywords:** progranulin, normal pressure hydrocephalus, prognostic factor, cerebrospinal fluid, serum, biomarker

## Abstract

**Background/Objectives:** Idiopathic normal pressure hydrocephalus (iNPH) is a potentially treatable syndrome, but biologically informative biomarkers remain limited. Progranulin (PGRN) constitutes a pleiotropic growth factor involved in neuroinflammation, lysosomal function, and tissue repair, which has not been adequately studied in iNPH. The purpose of this study was to examine the serum and cerebrospinal fluid (CSF) levels of PGRN in corresponding patients with suspected iNPH and its correlation with integrated clinical, functional, and neuroradiological parameters. **Methods:** Thirteen patients with probable iNPH underwent an evaluation protocol, including clinical assessment, neuroradiological evaluation, Tap-test with concomitant gait analysis, and paired serum/CSF sampling. PGRN concentrations in biofluids were measured by ELISA. Correlation analyses were performed. Composite Tap-test response variable derived from quantitative gait-improvement indices was modeled using ridge-logistic regression with leave-one-out cross-validation. **Results:** In the between-group analyses, serum and CSF concentrations of PGRN were not correlated (r = −0.10, *p* = 0.74), suggesting that peripheral and intrathecal PGRN behave as non-redundant, compartment-specific readouts rather than as interchangeable measures of the same biological process. Higher CSF concentration of PGRN was nominally associated with older age (r = 0.69, *p* = 0.009) and with poorer turning-time improvement after the Tap-test (r = −0.62, *p* = 0.025), while serum concentration of PGRN showed no meaningful associations with clinical or neuroradiological variables. In the model of logistic regression, inclusion of CSF concentration of PGRN substantially improved discrimination of Tap-test response. The full ridge-logistic regression model, including serum and CSF concentration of PGRN, symptom duration, and Kiefer score, achieved an accuracy of 0.923 and an AUC of 0.881. The CSF concentration of PGRN coefficient remained consistently negative across bootstrap resamples (penalized OR 0.434; 95% CI: 0.354–0.697), indicating that higher baseline CSF concentration of PGRN was associated with a lower probability of significant short-term Tap-test response, whereas serum PGRN contributed negligibly to the model. **Conclusions:** The observed changes in PGRN in CSF may reflect compartment-specific intrathecal inflammatory or tissue-stress processes and may help identify patients with lower short-term responsiveness to CSF drainage. These findings support further longitudinal evaluation of CSF concentration of PGRN for biological stratification and prognostic refinement in iNPH.

## 1. Introduction

Normal pressure hydrocephalus (NPH) is a clinical condition defined by gait disturbances, cognitive impairment, and urinary incontinence (Hakim–Adams triad) associated with neuroradiological features of ventriculomegaly, as well as seemingly normal intracranial pressure (ICP) [1]. NPH is typically classified into two types: idiopathic NPH (iNPH), when the underlying cause is unknown, and secondary NPH (sNPH), which can be caused by a variety of diseases such as subarachnoid hemorrhage (SAH), traumatic brain injury (TBI), intracranial malignancies, meningoencephalitis, or other cerebrovascular diseases [2]. Taking into consideration available large population-based studies, the prevalence of iNPH has been estimated to be 10–22 per 100,000 people overall, with 1.3% in those aged ≥65 years and 5.9% in those aged ≥80 years [3,4]. Therefore, regarding the retrospective nationwide population-based study of NPH cases in Germany from 2005 to 2022, the population-adjusted incidence of NPH increased by 48%, from 5.4 to 8.0 cases per 100,000 individuals, and was observed particularly affecting the elderly, underscoring its socioeconomic and health burden [5]. While the precise etiology of iNPH is still unknown, shunting is the principal treatment, predominantly utilizing ventriculoperitoneal (VP) shunts, which have been proven to be effective in alleviating symptoms in ~70% of cases [6,7]. Unfortunately, the persistent diagnostic uncertainty is clinically consequential, because iNPH remains one of the very few conditions in neurology and neurosurgery for which timely recognition may translate into meaningful functional improvement after cerebrospinal fluid (CSF) diversion [8]. Contemporary diagnostic frameworks of iNPH rely on the integration of clinical examination, neuroradiological imaging and CSF dynamic testing, yet none of these domains in isolation provides sufficient specificity for biologically grounded stratification [9]. To date, the specific pathogenesis and course of chronification of iNPH remains unclear, although several theories and pathophysiological mechanisms have been proposed [10]. Therefore, iNPH is no longer adequately understood as a purely hydrodynamic disorder characterized by “normal” CSF pressure despite ventricular enlargement [11]. Instead, the recent literature increasingly supports a multifactorial pathobiological model involving abnormal CSF circulation, impaired glymphatic system function, ependymal dysfunction, white matter vulnerability, astroglial and microglial activation, neuroimmunological reaction, blood–brain barrier (BBB) dysfunction, and frequent coexistence of age-related neurodegenerative pathology [12]. Large-scale CSF proteomic analyses have identified molecular signatures suggestive of altered synaptic homeostasis, disturbed cell-adhesion pathways, and ependymal or transependymal dysfunction, whereas inflammatory profiling studies have demonstrated that selected cytokine patterns may differentiate iNPH from other neurodegenerative disorders with clinically meaningful accuracy [13,14]. Collectively, these data indicate that the syndrome cannot be exhaustively captured by macroscopic ventricular geometry alone and that molecular readouts may be required to resolve the underlying biological heterogeneity of patients presenting with suspected iNPH. Recent biomarker-oriented literature has focused predominantly on amyloid-related analytes, tau proteins, markers of axonal injury, glial activation, neuroinflammation, and extracellular matrix remodeling with the dual aim of improving differential diagnosis and refining prognostic stratification [12,13]. Yet, despite increasingly sophisticated biomarker panels, no single analyte has emerged as sufficiently robust to define disease probability, grade symptom burden, or reliably forecast shunt responsiveness across diverse clinical settings. This limitation is particularly important in patients with incomplete triadic expression, prolonged symptom evolution, equivocal Tap-test response, or radiological ventricular morphometry findings that are suggestive but not decisive. Under such circumstances, a biomarker of genuine translational value would be expected to correlate with clinically interpretable parameters such as symptom time, gait–cognitive–urinary phenotype, quality of life, cognitive performance, and neuroradiological features. In recent years, the pleiotropic and multifunctional growth factor progranulin (PGRN) has emerged as a significant focus of interest in neuroscience research owing to its pronounced neurotrophic, anti-inflammatory, and immunomodulatory properties [15]. PGRN is a secreted glycoprotein composed of 593 amino acid residues and has a molecular weight of approximately 68.5 kDa in its primary form; following secretion, glycosylation increases its molecular mass to around 88 kDa [16]. Ultrastructurally, the protein is characterized by a series of 7.5 tandemly arranged granulin (GRN) domains that contain a conserved cysteine-rich motif with 12 residues (CX_5–6_CX_5_CCX_8_CCX_6_CCXDX_2_HCCPX_4_CX_5–6_C) [17]. These domains are organized in a specific order: p–G–F–B–A–C–D–E (p–P1–G–P2–F–P3–B–P4–A–P5–C–P6–D–P7–E), among them, domains A through G represent complete repeats, whereas the p domain constitutes a half-repeat motif. Current translational literature indicates that dysregulated PGRN expression is tightly linked to neurodegenerative disease biology, microglial state transitions, endolysosomal integrity, and neuroinflammatory signaling [18]. This mechanistic profile is particularly attractive in iNPH, a disorder increasingly interpreted through the lens of disturbed clearance (glymphatic) pathways, chronic subcortical tissue stress, and mixed neuroinflammatory–neurodegenerative processes [19]. From this perspective, PGRN is not simply another inflammatory molecule measurable in biofluids; rather, it is a candidate marker situated at the intersection of innate immunity, lysosomal competence, and neural resilience, which may be especially relevant in disorders where chronic CSF circulation abnormalities coexist with age-related tissue vulnerability. However, interpretation of PGRN in biofluids requires careful compartment-specific thinking, where a recent systematic review encompassing more than 7000 individuals demonstrated that PGRN concentrations could be influenced by biofluid compartment, assay platform, age, sex, genotype, and diagnostic category, arguing strongly against any simplistic assumption that serum and CSF presence of PGRN are interchangeable measures of the same biological process [20]. In this case, PGRN in serum may, at least in part, reflect peripheral inflammatory or metabolic context, whereas PGRN in CSF may more directly mirror intrathecal lysosomal and microglial biology. This distinction is further supported by recent longitudinal work in Alzheimer’s disease, in which PGRN in CSF varied across biological disease states and tracked neuropathological dynamics over time, suggesting that PGRN in CSF is biologically responsive rather than analytically inert [21]. Previous biofluid studies of PGRN have mainly used immunoassay-based approaches to quantify PGRN in plasma, serum and CSF. These studies indicate that PGRN values cannot be interpreted independently of the biological compartment and analytical platform used, because absolute concentrations and disease associations may vary according to sample type, assay configuration, calibration, age, sex, genotype, and diagnostic category [20]. Therefore, studies evaluating PGRN as a candidate biomarker should explicitly report the biofluid compartment and detection principle and should avoid direct biological equivalence between serum and CSF measurements unless this is empirically demonstrated. To date, the role of PGRN and its potential utility as a biomarker have not been extensively explored in the context of iNPH. Nevertheless, existing studies describe PGRN-associated neuroinflammatory and neurodegenerative processes that have been thoroughly investigated in preclinical rodent models [22,23]. In order to provide an accurate and current overview of the issue, the present study was designed to evaluate PGRN concentrations in serum and CSF in patients undergoing diagnostic work-up for suspected iNPH and to explore their relationships with the multidimensional clinical and radiological phenotype of the disorder. By integrating biofluid PGRN measurements with integrated clinical, functional, cognitive, and neuroradiological data, we sought to determine whether PGRN may serve as a plausible biomarker of disease burden and phenotypic architecture in iNPH, and whether serum and CSF provide complementary rather than redundant information in this diagnostically challenging syndrome.

## 2. Material and Methods

### 2.1. Patients and Clinical Analysis

The study and subsequent analyses were conducted on 13 (*n* = 13) patients recruited as outpatients or inpatients at the Department of Neurosurgery of the Dietrich-Bonhoeffer-Klinikum in Neubrandenburg – Academic Teaching Hospital of the Universitätsmedizin Greifswald (Table 1). The cohort covered 8 males (61.5%) and 5 females (38.5%) with a mean age of 76.6 ± 6.1 years (range: 64.5–83.6 years) and a mean body mass index (BMI) of 28.3 ± 4.1 kg/m^2^. Median symptom duration (SYMD) was 12.0 months (IQR: 12.0–18.0 months; range: 6–36 months). The diagnosis of iNPH was established according to the joint guidelines of the German Society of Neurology (DGN) and the German Society of Neurosurgery (DGNC) based on the presence of the following: (a) gait disturbance, (b) mental deterioration, (c) urinary incontinence, and (d) enlarged ventricles on CT or MRI with an Evan’s index (maximal width of frontal horns/maximal width of inner skull) >0.30 or callosal angle (angle between the left and right lateral ventricles at the level of the posterior commissure, perpendicular to the AC-PC plane) <90.0° [24]. The clinical history and the neuroradiological examination of each patient were carefully evaluated in order to rule out from the present study those patients with suspected iNPH. Medical records were further verified using predefined, literature-based exclusion criteria. Exclusion criteria included consecutively neurological diseases inclusive frontotemporal lobar degeneration (FTLD), Alzheimer’s disease, encephalitis, meningitis, motor neuron disease, Parkinson’s disease, amyotrophic lateral sclerosis (ALS), multiple sclerosis (MS), Creutzfeldt-Jakob disease, neuronal ceroid lipofuscinosis, epilepsy, cerebrovascular disease, schizophrenia and bipolar disease, as well as non-neurological states and conditions including another inflammatory, rheumatoid or autoimmune diseases, diabetes mellitus, hepatic insufficiency and disease, renal insufficiency and disease, prior cancer (malignancy) history, adiposity, cachexia, malnutrition, history of acute trauma or surgery within 6 months, history of infectious disease within 6 months, as well as therapy with drugs that potentially affect epigenetic pathways including glucocorticoids, histone deacetylase (HDAC) inhibitors and selective serotonin reuptake inhibitors (SSRI). A strict selection was implemented with the assumption that changes in PGRN expression and concentration level in tissues and biofluids are potentially related to the above-mentioned conditions. All patients were previously thoroughly acquainted with the conditions of the study and signed individual informed consent regarding their voluntary participation. This study was approved by the Ethics Commission of the Universitätsmedizin Greifswald (BB 015/24, approved on 19 April 2024). All procedures were performed in accordance with the Declaration of Helsinki (DoH) and its later amendments, as well as in compliance with the Good Clinical Practice (GCP) guidelines, including combining Federal Republic of Germany legislation with European Commission (EC) directives in this area. The individual data of each patient covered anthropometric and demographic variables (sex, age, BMI), as well as clinical variables such as SYMD, elements of Hakim–Adams triad, Tap-test evaluation parameters, Kiefer score (KS), WHOQOL-BREF score, MMSE score, and decision regarding VP shunt implantation. Further data covered neuroradiological parameters (Evans index, ALVI index, third ventricle width, callosal angle, temporal horn width). Consecutively, the blood and serum collected before the Tap-test was examined routinely for red blood cells (RBC^BLOOD^), leukocytes (LEU^BLOOD^), hemoglobin (HGB), platelets (PLT), C-reactive protein (CRP), interleukin-6 (IL-6^SERUM^), as well as PGRN (PGRN^SERUM^). The Tap-test was performed using a standardized lumbar puncture protocol with removal of ~40 mL of CSF under sterile conditions. Quantitative gait assessment was conducted immediately prior to the procedure and repeated 24 h post-intervention. The evaluation protocol included measurement of the number of steps and time required to traverse a 10 m distance, as well as the number of steps and time necessary to complete a 360° turn. All evaluations were performed under standardized conditions using an identical protocol to ensure intra-individual comparability and reproducibility. The obtained CSF was examined routinely for physical appearance, LEU^CSF^, RBC^CSF^, total protein, glucose, IL-6^CSF^, lactate, as well as PGRN (PGRN^CSF^).

### 2.2. Biofluid Specimens Obtainment and Preparation

The CSF samples were obtained through lumbar puncture (LP) during the Tap-test procedure by a senior neurosurgeon. Patients were placed in the lateral decubitus position with the hips and knees flexed to maximize interspinous space exposure. The puncture site was identified by palpating anatomical landmarks, typically at the L3/L4 or L4/L5 intervertebral space, corresponding to the level of the iliac crest (Tuffier’s line). After skin disinfection with an alcohol-based antiseptic solution, the puncture site was draped using sterile technique. Local anesthesia was administered by infiltrating 1% lidocaine (C_14_H_22_N_2_O) into the skin and subcutaneous tissue. A sterile spinal needle (typically 20–22 G) with stylet was inserted in the midline and advanced slowly through the supraspinous ligament, interspinous ligament, and ligamentum flavum until entry into the subarachnoid space was achieved, as indicated by the free flow of CSF upon removal of the stylet. For analyses, CSF was collected according to a prespecified pre-analytical protocol directly into sterile low-binding RNA/DNAse-free polypropylene cryotubes, with sample transfers kept to a minimum. Typically, ~40 mL (4 × 10 mL) of CSF was obtained and distributed into separate tubes for analyses. Following collection, samples were gently mixed, visually inspected for blood contamination, and processed without unnecessary delay. After sample collection, the stylet was reinserted, and the needle was carefully withdrawn. Gentle pressure was applied to the puncture site using sterile gauze, followed by placement of a sterile dressing. Patients were monitored for procedure-related complications, including post-dural puncture headache, bleeding, or neurological symptoms. All procedures were conducted in accordance with institutional clinical protocols and standard safety guidelines. The CSF samples prespecified for PGRN^CSF^ analysis were processed at 1600 rpm for 10 min to obtain the supernatant. After processing, CSF was aliquoted into single-use low-binding polypropylene cryovials and stored at −80 °C until batch analysis. Freeze–thaw cycles were minimized, and all pre-analytical variables, including time from collection to processing/freezing and storage conditions, were standardized across participants. The CSF samples were prespecified for routine examination analysis, and the control microbiological examinations were processed according to the standard internal laboratory and microbiological procedures. Venous blood was collected before the Tap-test by standard venipuncture into serum collection tubes. After collection, PGRN^SERUM^-prespecified tubes were gently inverted according to the manufacturer’s recommendations, kept upright at room temperature for 30–60 min to allow clot formation, and centrifuged at 6000 rpm for 10 min to separate the serum. Serum was then transferred carefully to avoid disturbing the clot, gently mixed, aliquoted into low-binding polypropylene cryovials, and stored at −80 °C until analysis. Freeze–thaw cycles were minimized. Any samples that showed visible signs of hemolysis were excluded from testing. The blood samples prespecified for routine analysis were processed according to the standard internal laboratory procedures.

### 2.3. ELISA

**Figure 1:**
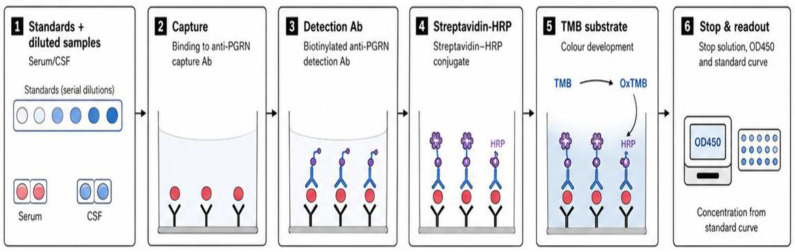
Technical principle of the double-antibody sandwich ELISA used for PGRN quantification in paired serum and CSF samples.

Analysis of the presence and concentration level of PGRN in sampled body liquids (CSF and serum) was evaluated (according to the standardized manual supplied by the manufacturer) using an enzyme-linked immunosorbent assay (ELISA) test. A series of assays was carried out using the commercially available Human PGRN (Progranulin) ELISA Kit (EH4861; Wuhan Fine Biotech, Wuhan, China). Concisely, standards and samples were incubated in microplate wells precoated with anti-human PGRN antibody for 1.5 h, consecutively followed by 1 h incubation with a biotin-labeled monoclonal PGRN antibody solution and 30 min incubation with a streptavidin–horseradish peroxidase (HRP) conjugate at 37 °C (Figure 1). Between each step, the plate was emptied and thoroughly washed with a washing buffer solution according to the test manual. After the last washing step, the substrate solution 3,3′,5,5′-tetramethylbenzidine (C_16_H_20_N_2_) was added and incubated for a further 25 min. The reaction was stopped by the addition of an acidic solution to each well in the same order. Consequently, the following photometric assessment covering the absorbance of the resulting color product was measured using a microplate reader within 2 h at a wavelength (λ) of 450 nm. The interpretation of acquired PGRN concentration levels was determined using the standard curve. The following series of assays was tested in two subsequent repetitions, and the average results were used for analysis. The detection range for the test assay was 0.313–20 ng/mL; dilution rates for CSF were up to 1:20, and up to 1:30 for serum.

### 2.4. Statistical Analysis

The data were analyzed using the Python (version 3.12.12; Python Software Foundation, Wilmington, DE, USA) software package for Windows (Microsoft Corporation, Redmond, WA, USA). For this purpose, individual modules and sets of numerical algorithms included in the SciPy (version 1.17.0), Matplotlib (version 3.10.8), NumPy (version 2.3.5), and Pandas (version 2.3.3) libraries were used. At a preliminary stage of data processing, Microsoft Office Excel 2010 (Microsoft Corporation, Redmond, WA, USA) was employed for data collection, variable annotation, basic visualization, and the calculation of simple descriptive statistics prior to subsequent, more thorough analysis. Normality of continuous variables was assessed using the Shapiro–Wilk test (significance threshold: *p* > 0.05). Bivariate correlations were computed using a method selected for each pair according to its measurement scale and distributional properties: Pearson’s r was applied when both variables were continuous and normally distributed; Spearman’s rank correlation coefficient (ρ) was applied for ordinal variables, non-normally distributed continuous variables, or mixed-type pairs; point-biserial correlation was applied for binary-continuous pairs; and Spearman’s ρ for binary-ordinal pairs. Correlation analyses were performed within each of the eight predefined data groups and between eleven group pairs; the pairs were specified on an a priori, hypothesis-driven basis in accordance with the study design and existing literature. Because all between-group comparisons were hypothesis-driven and pre-specified before data inspection, raw *p*-values are reported without correction for multiple comparisons; application of false discovery rate (FDR) or family-wise error correction to pre-specified, hypothesis-grounded analyses in a sample of this size would substantially reduce power without a commensurate reduction in type I error risk. All associations are interpreted as exploratory findings requiring independent replication. Statistical significance was set at α = 0.05; associations with 0.05 ≤ *p* < 0.10 are noted as statistical trends (†). A composite Tap-test response variable (TTRV) was constructed from four percentage-improvement indices: walking time (TTWTi), walking steps (TTWSi), turning time (TTTTi), and turning steps (TTTSi) using principal component analysis (PCA) applied to z-standardized indices. The first principal component (PC1) was retained as TTRV because its eigenvalue exceeded unity (λ_1_ = 3.08, explaining 71.0% of total variance) and all four PC1 loadings were uniformly positive (range 0.461–0.541), confirming PC1 as a unidimensional global Tap-test responsiveness factor. TTRV was dichotomized at its sample median to yield a binary outcome variable (0 = little/no response, 1 = significant response; n_0_ = 6, n_1_ = 7). For logistic regression modeling of Tap-test response, five candidate predictors were screened for multicollinearity. The number of elements of Hakim–Adams triad was excluded due to high collinearity with KS (Pearson r = 0.826); the remaining four predictors, such as PGRN^CSF^ (as mandatory biomarker), PGRN^SERUM^, SYMD, and KS, were retained. Standard maximum likelihood logistic regression failed to converge due to complete separation of the outcome classes. For prediction of Tap-test response, L2-penalized (Ridge) logistic regression was adopted, with optimal regularization strength C = 0.5 selected by leave-one-out cross-validation (LOOCV), achieving a peak accuracy of 0.923. Coefficient uncertainty was quantified by 95% bootstrap confidence intervals (2000 resamples, percentile method). Model performance was evaluated by LOOCV accuracy, area under the receiver operating characteristic (ROC) curve (AUC), sensitivity, and specificity.

## 3. Results

### 3.1. Study Variables and Data Structure

The analysis dataset comprised 13 patients (*n* = 13) and 32 variables assigned in predefined groups covering anthropometric and demographic variables, clinical variables, neuroradiological parameters, blood serum markers, and CSF markers. The dataset contains variables spanning at least four distinct measurement levels (binary categorical, ordinal psychometric, continuous ratio-scale, and count-like laboratory). The performed Shapiro–Wilk testing revealed that laboratory markers (serum and CSF) were predominantly non-normally distributed, while neuroradiological parameters, Tap-test improvement parameters, and demographic variables were normally distributed.

### 3.2. Intra-Group Correlations

#### 3.2.1. Clinical Variables Including Anthropometric and Demographic Data

**Figure 2:**
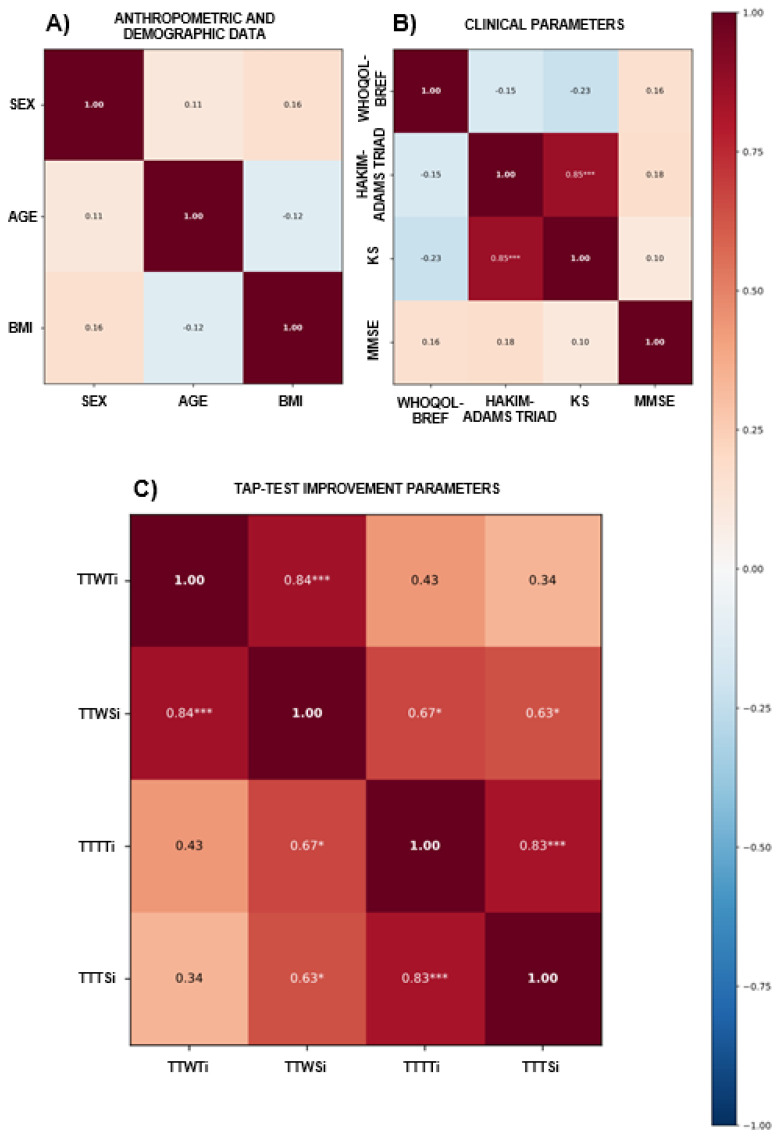
The intra-group correlation matrices for anthropometric and demographic data (**A**), clinical parameters (**B**) and Tap-test improvement parameters (**C**). Color scale: red = positive, blue = negative correlation. Annotations show the correlation coefficient and uncorrected significance: * *p* < 0.05, *** *p* < 0.001.

No significant correlations were found among sex, age, and BMI (all *p* > 0.60), confirming the independence of anthropometric and demographic variables from each other (Figure 2A). Presence of the Hakim–Adams triad elements and KS results were strongly correlated (ρ = 0.85, *p* < 0.001, FDR *p* = 0.001), confirming convergent validity between these two instruments that both measure clinical severity of hydrocephalus (Figure 2B). WHOQOL-BREF score showed no significant correlation with the presence of the Hakim–Adams triad elements, KS and MMSE (all *p* > 0.46), indicating that patient-reported quality of life captures a distinct dimension from clinician-rated symptom burden. The four gait improvement parameters showed strong internal coherence. TTWTi and TTWSi were highly correlated (ρ = 0.84, *p* < 0.001, FDR *p* = 0.002), as were TTTTi and TTTSi (r = 0.83, *p* < 0.001), demonstrating that each domain pair captures a common underlying factor (Figure 2C). TTWSi also correlated significantly with TTTTi (ρ = 0.67, *p* = 0.012) and TTTSi (ρ = 0.63, *p* = 0.020). The cross-domain pair TTWTi-TTTTi did not reach significance (r = 0.43, *p* = 0.143), suggesting some independence between the straight-walking and turning components of the Tap-test gait analysis.

#### 3.2.2. Neuroradiological Parameters

**Figure 3:**
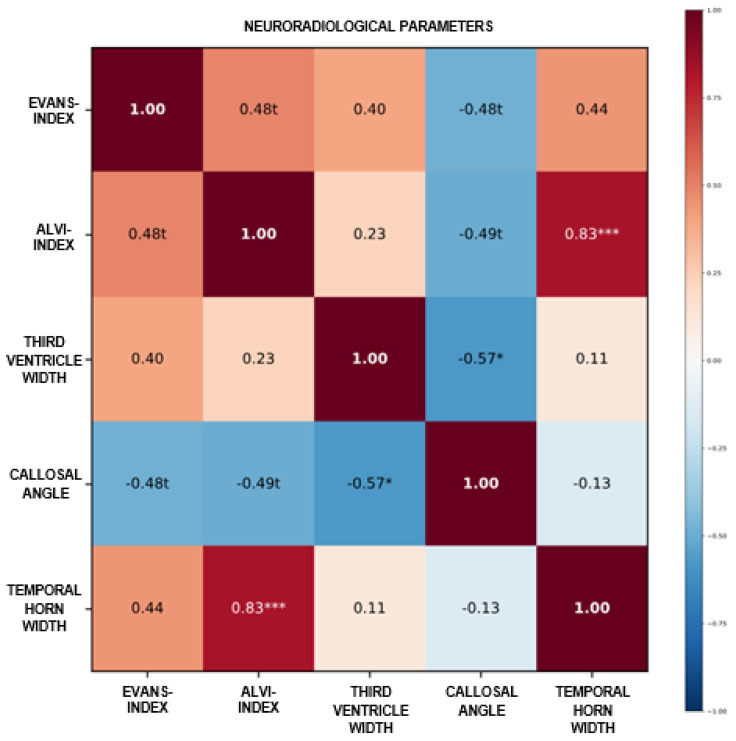
The intra-group correlation matrices for neuroradiological parameters. Color scale: red = positive, blue = negative correlation. Annotations show the correlation coefficient and uncorrected significance: * *p* < 0.05, *** *p* < 0.001.

ALVI index and temporal horn width co-varied strongly (r = 0.83, *p* < 0.001, FDR *p* = 0.005), reflecting concurrent enlargement of the lateral ventricular structures (Figure 3). Third ventricle width was negatively correlated with callosal angle (r = −0.57, *p* = 0.042), consistent with the known biomechanical inverse relationship between ventricular dilation and corpus callosum angulation in iNPH. Evans index showed trends toward correlation with ALVI index (r = 0.49, *p* = 0.093) and callosal angle (r = −0.48, *p* = 0.099) but neither survived FDR correction.

#### 3.2.3. Blood and Serum Markers

**Figure 4:**
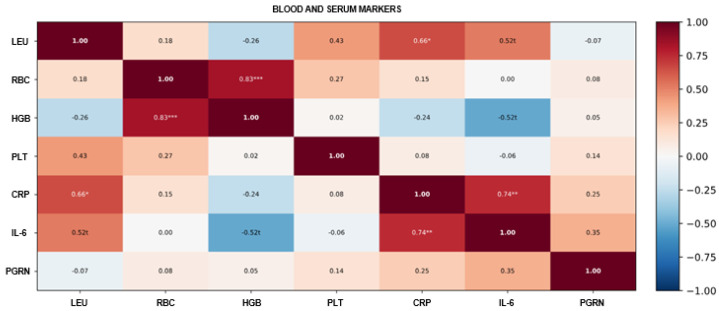
The intra-group correlation matrices for blood and serum markers. Color scale: red = positive, blue = negative correlation. Annotations show the correlation coefficient and uncorrected significance: * *p* < 0.05, ** *p* < 0.01, *** *p* < 0.001.

The strongest blood and serum association was between RBC^BLOOD^ and HGB (r = 0.83, *p* < 0.001, FDR *p* = 0.008), a well-established physiological coupling (Figure 4). CRP and IL-6^SERUM^ were significantly correlated (ρ = 0.74, *p* = 0.004, FDR *p* = 0.037), reflecting shared acute-phase inflammatory signaling. LEU^BLOOD^ showed a significant association with CRP (ρ = 0.66, *p* = 0.015), consistent with leukocytosis accompanying elevated CRP. PGRN^SERUM^ was not significantly correlated with any other serum marker (all *p* > 0.24), suggesting it varies independently from conventional inflammatory parameters in this cohort.

#### 3.2.4. CSF Markers

**Figure 5:**
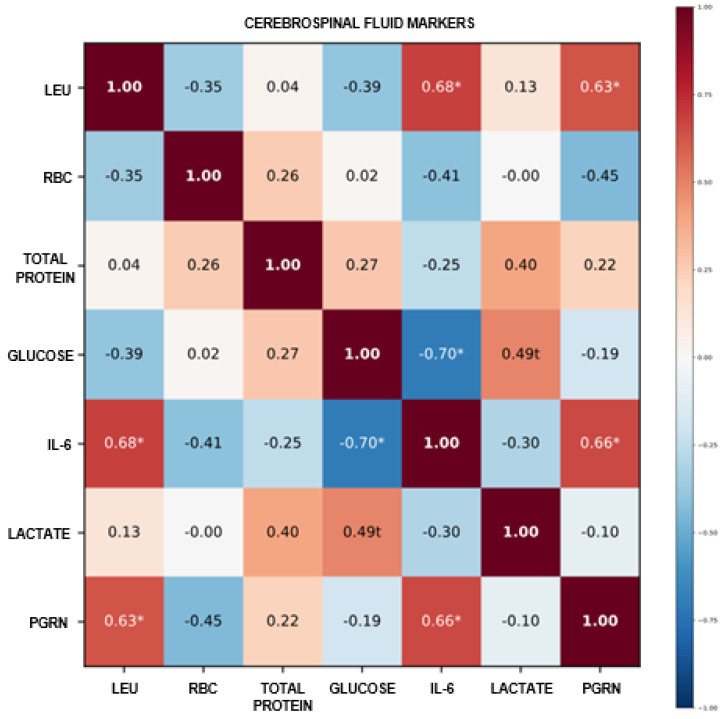
The intra-group correlation matrices for cerebrospinal fluid markers. Color scale: red = positive, blue = negative correlation. Annotations show the correlation coefficient and uncorrected significance: * *p* < 0.05.

Notable within-group associations were observed between LEU^CSF^ and IL-6^CSF^ (ρ = 0.68, *p* = 0.021) and between LEU^CSF^ and PGRN^CSF^ (ρ = 0.63, *p* = 0.021), suggesting that CSF cellular and molecular inflammatory activity are inter-related (Figure 5). Glucose and IL-6^CSF^ were negatively correlated (ρ = −0.70, *p* = 0.016), consistent with glucose consumption under inflammatory conditions. Importantly, PGRN^CSF^ also correlated positively with IL-6^CSF^ (ρ = 0.66, *p* = 0.026), pointing to a possible involvement of PGRN in the CSF neuroinflammatory reaction occurring in the iNPH course.

### 3.3. Between-Group Correlations

#### 3.3.1. Clinical Variables Including Anthropometric and Demographic Data

**Figure 6:**
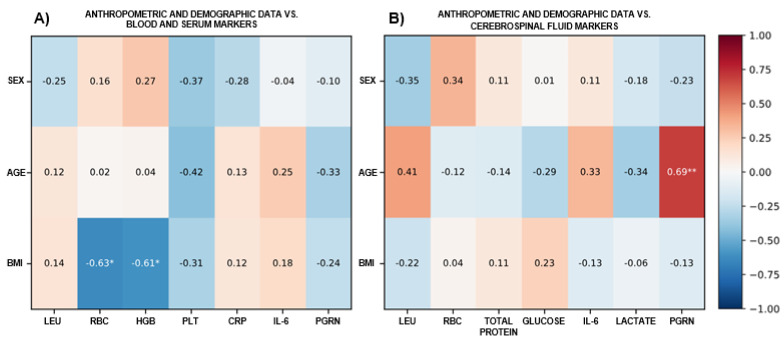
The between-group correlation matrices for anthropometric and demographic data vs. blood and serum markers (**A**) and anthropometric and demographic data vs. cerebrospinal fluid markers (**B**). Color scale: red = positive, blue = negative correlation. Annotations show the correlation coefficient and significance: * *p* < 0.05, ** *p* < 0.01.

**Figure 7:**
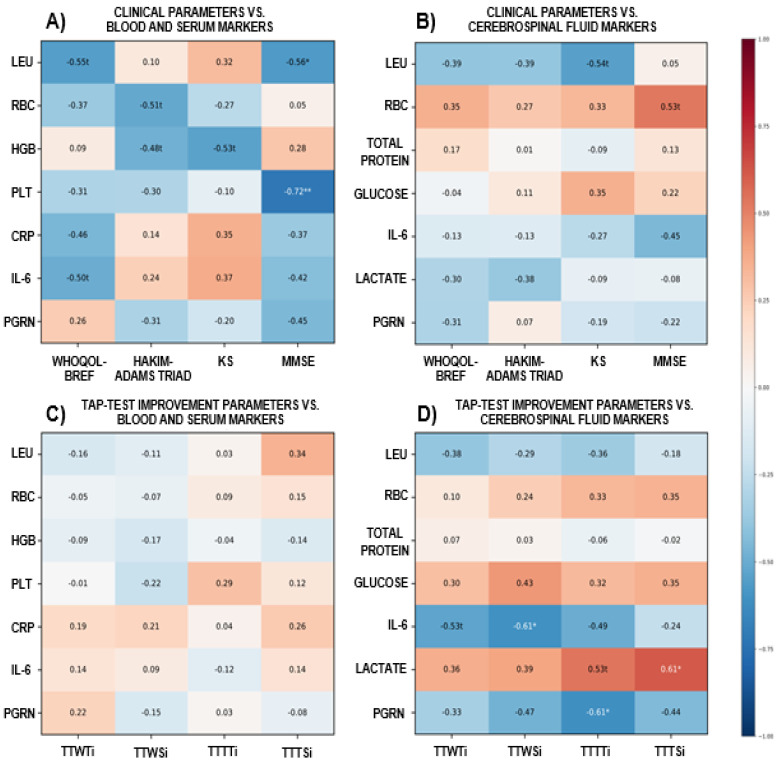
The between-group correlation matrices for clinical parameters vs. blood and serum markers (**A**), clinical parameters vs. cerebrospinal fluid markers (**B**), Tap-test improvement parameters vs. blood and serum markers (**C**), and Tap-test improvement parameters vs. cerebrospinal fluid markers (**D**). Color scale: red = positive, blue = negative correlation. Annotations show the correlation coefficient and significance: * *p* < 0.05, ** *p* < 0.01.

Age was significantly positively correlated with PGRN^CSF^ (r = 0.69, *p* = 0.009), the strongest association in the demographics matrix; the magnitude and direction of this association are consistent with published literature on age-related PGRN dysregulation, and age was incorporated as a consideration in the regression modeling design (Figure 6B). No significant associations were found between sex or BMI and any CSF or blood and serum marker (Figure 6A,B). Three nominally significant CSF–Tap-test associations were identified (Figure 7D). PGRN^CSF^ was negatively correlated with TTTTi (r = −0.62, *p* = 0.025), indicating that patients with higher baseline PGRN^CSF^ showed less improvement in this motor parameter following CSF drainage. IL-6^CSF^ was negatively correlated with TTWSi (ρ = −0.61, *p* = 0.048), and the CSF concentration level of lactate was positively correlated with TTTSi (ρ = 0.61, *p* = 0.026).

#### 3.3.2. Neuroradiological Parameters

**Figure 8:**
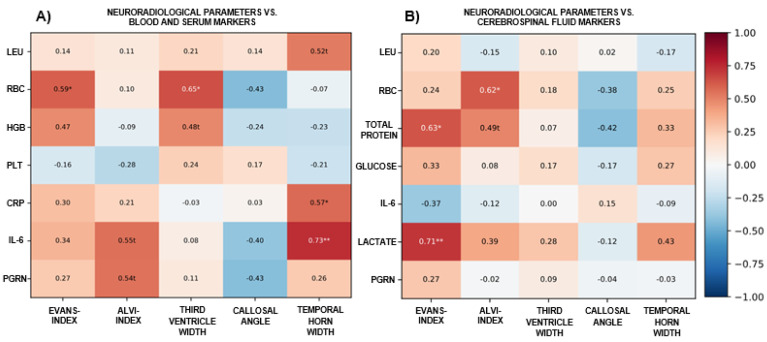
The between-group correlation matrices for neuroradiological parameters vs. blood and serum markers (**A**) and neuroradiological parameters vs. cerebrospinal fluid markers (**B**). Color scale: red = positive, blue = negative correlation. Annotations show the correlation coefficient and significance: * *p* < 0.05, ** *p* < 0.01.

Several nominally significant associations between laboratory markers and ventricular morphology were observed. IL-6^SERUM^ correlated positively with temporal horn width (ρ = 0.74, *p* = 0.004), suggesting that higher serum inflammatory load may accompany greater lateral ventricular enlargement (Figure 8A). CSF concentration levels of lactate and total protein correlated with Evans index (ρ = 0.72, *p* = 0.006 and r = 0.63, *p* = 0.021, respectively), linking CSF metabolic and inflammatory parameters to the extent of ventricular dilation (Figure 8B).

#### 3.3.3. Blood, Serum and CSF Markers

No significant cross-compartment correlations were found. PGRN^SERUM^ and PGRN^CSF^ were not correlated (r = −0.10, *p* = 0.74). We therefore treated serum and CSF levels of PGRN as non-redundant readouts rather than as interchangeable measurements of the same biological process. Biologically, this lack of association is plausible because PGRN^SERUM^ may reflect peripheral inflammatory, metabolic, leukocyte- or platelet-related, and systemic tissue-repair signals, whereas PGRN^CSF^ is more likely to reflect intrathecal microglial, endolysosomal, and neuroinflammatory activity. This compartment-specific interpretation is consistent with the broader PGRN biofluid literature, which indicates that measured PGRN concentrations depend on biofluid compartment and assay-related factors. The absence of a serum–CSF correlation also provided a statistical rationale for including PGRN^SERUM^ and PGRN^CSF^ as separate candidate predictors in the regression model. A nominally significant association between RBC^BLOOD^ and CSF concentration level of lactate (ρ = 0.59, *p* = 0.034) was also observed.

### 3.4. Development and Evaluation of Predictive Tap-Test Response Model Including PGRN

#### 3.4.1. Tap-Test Composite and Logistic Regression Modeling

**Figure 9:**
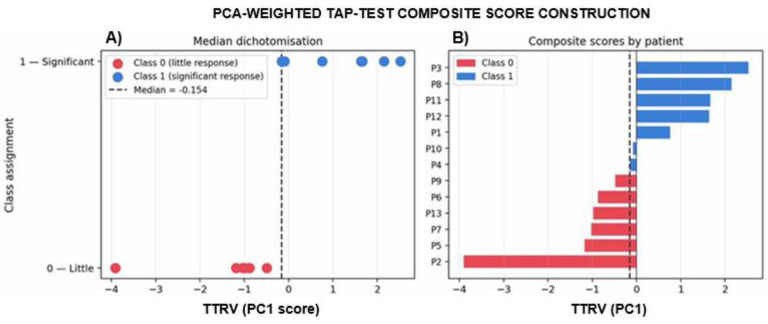
PCA-weighted Tap-test composite score construction. (**A**) Scatter of TTRV (PC1) values by class with median cut-off (dashed line, −0.154) and (**B**) ranked bar chart of individual patient composite scores colored by class assignment (class 0 = little response; class 1 = significant response).

**Figure 10:**
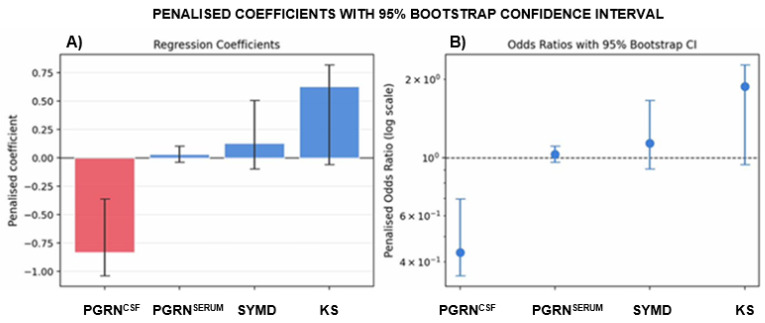
The penalized logistic regression coefficients with 95% bootstrap confidence intervals (CI) of 2000 resamples. (**A**) Bar chart of penalized regression coefficients and (**B**) penalized odds ratios on log scale. PGRN^CSF^ is the only predictor whose 95% CI lies entirely below zero (coefficient scale) and entirely below 1.0 (odds ratio scale).

To model the probability of significant Tap-test response, the four improvement indices were integrated into a composite outcome using PCA. The first principal component (PC1; TTRV) explained 71.0% of total variance (λ_1_ = 3.076; λ_2_ = 0.968, below the Kaiser threshold of 1.0), with uniformly positive loadings across all four indices (TTWSi: 0.541; TTTTi: 0.518; TTTSi: 0.476; TTWTi: 0.461), confirming PC1 as a unidimensional global responsiveness factor. TTRV was dichotomized at its sample median to yield a balanced binary outcome (Tap-test binary response score: n_0_ = 6, n_1_ = 7). Notably, binary outcome labels were identical to those produced by equal-weight averaging of the four standardized indices (Pearson r = 0.9999; 100% label concordance), validating the robustness of the classification with respect to the weighting scheme. The distribution of TTRV values and resulting class assignments is illustrated in Figure 9. Predictor selection was informed by the correlation analyses. The confirmed independence of PGRN^CSF^ and PGRN^SERUM^ (r = −0.10) supported their simultaneous inclusion as distinct predictors. The four retained predictors were PGRN^CSF^ (mandatory biomarker), PGRN^SERUM^, SYMD and KS. Standard maximum-likelihood logistic regression resulted in complete separation; the predictor combination perfectly classified all 13 observations, preventing finite coefficient estimation. This outcome, while precluding standard inferential procedures, provides initial evidence of strong discriminative capacity of the predictor combination. For prediction of Tap-test response, L2-penalized (Ridge) logistic regression was adopted, with the optimal regularization strength (C = 0.5) selected by LOOCV, achieving a peak accuracy of 0.923 in the analyzed cohort (Figure 10). The linear predictor (log-odds) is given by

η = β_0_ + β_1_ · PGRN^CSF^ + β_2_ · PGRN^SERUM^ + β_3_ · SYMD + β_4_ · KS

where β_0_ = −0.001 (intercept), β_1_ = −0.834, β_2_ = 0.032, β_3_ = 0.130, β_4_ = 0.632 (Table 2).

#### 3.4.2. Model Performance and Incremental Contribution of CSF Concentration Level of PGRN

**Figure 11:**
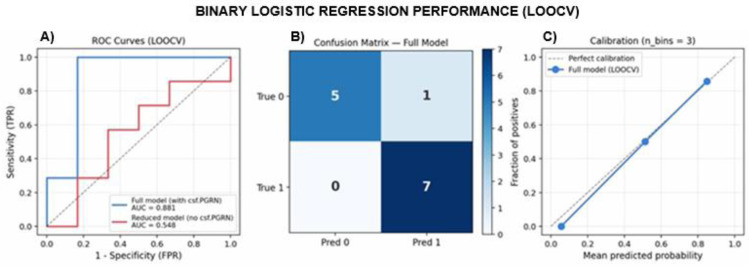
The binary logistic regression model performance (LOOCV). (**A**) ROC curves for the full model (with PGRN^CSF^, AUC = 0.881, blue) and the reduced model (without PGRN^CSF^, AUC = 0.548, red); dashed diagonal = chance level. (**B**) LOOCV confusion matrix for the full model. (**C**) Calibration curve (n_bins = 3); dashed line = perfect calibration.

The full model (including PGRN^CSF^) achieved an accuracy of 0.923 (12/13 correct classifications), AUC of 0.881, sensitivity of 1.000, and specificity of 0.833 (Table 3). When PGRN^CSF^ was excluded, model performance decreased markedly to an accuracy of 0.538 and AUC of 0.548, near the level of random classification. The incremental contribution of PGRN^CSF^ to discriminative performance (ΔAUC = +0.333) confirms it as the key driver of Tap-test response discrimination within this predictor combination. The implemented LOOCV and ROC curves, confusion matrix, and calibration plot are shown in Figure 11. The PGRN^CSF^ coefficient was consistently negative across all 2000 bootstrap resamples, with a penalized odds ratio (OR) of 0.434 (95% CI: 0.354–0.697), entirely below 1.0, indicating that higher baseline PGRN^CSF^ is associated with a lower probability of significant Tap-test motor response. In contrast, PGRN^SERUM^ contributed negligibly (OR 1.033; 95% CI crossing null). KS showed a positive trend (OR 1.881) and symptom duration a modest positive association (OR 1.139), both with wide confidence intervals reflecting the exploratory nature of these estimates. Calibration was assessed across three LOOCV probability bins and demonstrated reasonable correspondence between predicted probabilities and observed event rates (low bin: predicted 0.055, observed 0.000; medium bin: 0.513, observed 0.500; high bin: 0.848, observed 0.857).

## 4. Discussion

The present study investigated the associations between PGRN^CSF^ and PGRN^SERUM^ and a comprehensive set of clinical, functional, cognitive, and neuroradiological parameters in patients undergoing a diagnostic protocol for iNPH. Accordingly, we present preliminary but conceptually important evidence that PGRN measured in CSF and serum should not be regarded as interchangeable readouts in the case of iNPH. The key finding and relevant observation emerging from our dataset is the biological asymmetry between these two compartments: whereas PGRN^SERUM^ showed no meaningful relationship with conventional systemic inflammatory variables or with the PGRN^CSF^, intrathecal PGRN was linked to LEU^CSF^, IL-6^CSF^, age, and, most importantly from a clinical perspective, to short-term motor responsiveness after large-volume mechanical CSF removal (Tap-test). In our exploratory model, PGRN^CSF^ was the only biomarker whose inclusion materially improved model discrimination, and its effect direction was consistently unfavorable, with higher baseline CSF concentrations associated with a lower probability of significant Tap-test response. These findings argue against a simplistic viewing and interpretation of PGRN as a generic inflammatory analyte and instead support its consideration as a compartment-specific marker of intrathecal tissue stress, neuroimmune activation, and possibly reduced reversibility of the altered CSF dynamics characteristic of iNPH. This interpretation fits with the recent view of iNPH as a syndrome that cannot be exhaustively reduced to altered CSF hydrodynamics alone [25]. Although ventricular enlargement and disturbed CSF circulation remain central to disease definition, contemporary work increasingly situates iNPH at the intersection of impaired glymphatic clearance, ependymal dysfunction, periventricular white matter vulnerability, cerebrovascular dysregulation, and low-grade neuroinflammatory signaling [26]. To date, comprehensive CSF proteomic investigations have delineated molecular signatures reflective of disturbed extracellular matrix homeostasis, aberrant cell adhesion, synaptic dysregulation, and ependymal pathology, whereas targeted inflammatory profiling indicates that discrete cytokine patterns may serve as discriminative biomarkers, distinguishing iNPH from prototypical neurodegenerative disorders [27]. In that context, the present results are notable because PGRN is mechanistically positioned at a particularly relevant crossroads: it is not only associated with inflammation in a broad sense, but is deeply implicated in lysosomal competence, microglial phenotype regulation, neuronal resilience, and tissue repair [28]. Therefore, such unique biology makes PGRN especially attractive in a condition in which chronic abnormalities of fluid circulation likely coexist with repeated microenvironmental stress at the ependymal and subependymal interface. In our studied iNPH cohort, PGRN^SERUM^ appeared biologically detached, showing no significant association with CRP, IL-6^SERUM^, or routine blood-cell indices. By contrast, PGRN^CSF^ clustered with intrathecal inflammatory measures. This division suggests that, at least under the stringent exclusion criteria applied in the present study, systemic inflammatory noise does not substantially account for PGRN^CSF^ variability. Instead, intrathecal PGRN appears to be linked to local neuroimmune or neuroglial phenomena. From a translational standpoint, this matters because it implies that a negative serum result cannot be used as a surrogate for the intrathecal biological state and that CSF-based assessment is likely indispensable if PGRN is to be evaluated as a clinically meaningful biomarker in iNPH. The positive correlations between PGRN^CSF^, IL-6^CSF^, and LEU^CSF^ count further support the hypothesis that PGRN participates in a low-grade neuroinflammatory milieu in iNPH [29]. Importantly, the studied patients were carefully screened to exclude overt infectious, systemic inflammatory, autoimmune, metabolic, medication-influenced, and major neurodegenerative confounders that are known to potentially perturb PGRN biology. These intrathecal associations are unlikely to be explained by nonspecific noncentral inflammatory influence. Rather, they are more plausibly interpreted as reflecting a sterile, chronic, CNS-restricted inflammatory tone. This interpretation is compatible with prior work suggesting that inflammatory signaling is not an incidental epiphenomenon in iNPH but may represent a constitutive element of disease biology [30]. The IL-6^CSF^ coupling is particularly interesting; its association with PGRN^CSF^ in our cohort may indicate that PGRN is induced as part of an endogenous counter-regulatory response to ongoing tissue stress. In other words, elevated PGRN^CSF^ in iNPH may not simply denote neuroprotection; it may instead mark the presence of a biological state in which compensatory anti-inflammatory and lysosomal-support pathways have already been mobilized in response to ongoing chronic injury [31]. This point is important because PGRN biology is fundamentally ambivalent when interpreted in cross-sectional clinical samples. Experimental and translational studies indicate that PGRN exerts neurotrophic and immunomodulatory effects, supports lysosomal function, influences microglial activation states, and may mitigate neurodegenerative damage [32,33]. Yet an increased concentration in biofluids does not necessarily signify biological sufficiency or effective protection [34]. On the contrary, elevated PGRN may also be understood as a reactive signal, emerging precisely where lysosomal burden, microglial activation, white matter injury, or impaired protein clearance are most pronounced [23]. In GRN-associated neurodegeneration, disturbed PGRN signaling promotes microglial dysfunction, lysosomal failure, and white matter pathology [35]. Although iNPH is not a confirmed PGRN-opathy, the conceptual parallel is informative: a chronic hydrocephalic environment characterized by impaired solute clearance, subependymal stress, and axonal vulnerability may trigger a similar axis of microglial-lysosomal compensation. Our finding that higher PGRN^CSF^ is associated with poorer short-term post-Tap-test motor improvement is consistent with that reading and suggests that increased PGRN may be less a marker of preserved compensatory reserve than a signature of tissue-level burden already substantial enough to limit immediate functional reversibility. The age relationship observed for PGRN^CSF^ also fits this interpretation. Age is the principal epidemiological risk substrate for iNPH, and aging itself is accompanied by microglial priming, altered lysosomal trafficking, reduced photostatic efficiency, and heightened vulnerability to chronic neuroinflammation [36]. In this context, the positive age-PGRN^CSF^ association may reflect the convergence of two processes: age-related “inflammaging” and disease-specific hydrocephalic stress. This is clinically relevant because it suggests that the biological meaning of PGRN^CSF^ in iNPH may not be uniform across all patients. A given concentration may encode a different pathological signal in a younger-old versus an older-old patient, especially when mixed neurodegenerative pathology is considered. Recent longitudinal work in Alzheimer’s disease has shown that PGRN^CSF^ tracks neuropathological dynamics over time rather than behaving as an inert background analyte [21]. Extrapolated cautiously to iNPH, this argues that PGRN may be biologically responsive to ongoing CNS pathology and therefore potentially useful not only for cross-sectional phenotyping but also for longitudinal monitoring in future studies. The strongest clinical implication of our findings lies in the association between higher PGRN^CSF^ and reduced Tap-test responsiveness, especially in turning performance, as well as in the dominant contribution of PGRN^CSF^ to the penalized logistic regression model. Although one must be careful not to overinterpret model-derived results in a limited cohort, the internal consistency of the signal is striking. Turning covers a particularly demanding motor task in iNPH because it taxes axial control, postural adaptation, sensorimotor integration, and frontal–subcortical executive–motor coordination more heavily than straightforward gait alone [37]. The negative relation between baseline PGRN^CSF^ and TTTTi therefore raises the possibility that elevated intrathecal PGRN identifies patients in whom the gait disorder is less purely hydrodynamic and more strongly shaped by chronic white matter or subcortical network injury. This distinction is clinically consequential; if confirmed, PGRN^CSF^ would not simply serve as another routine biomarker, but rather as a stratification tool capable of identifying a subgroup with lower short-term functional plasticity after CSF drainage. This role would fit well within the current clinical reality of iNPH management, where Tap-test results, although widely used, are imperfect and may be equivocal in patients with long symptom duration, incomplete triadic expression, or mixed comorbidity [38]. Likewise, neuroradiological parameters (Evans index, ALVI index, third ventricle width, callosal angle, temporal horn width) are diagnostically useful but biologically coarse; they quantify ventricular geometry rather than the intraparenchymal response to chronic hydrocephalic stress [39]. In our cohort, the neuroradiological parameters were internally coherent and behaved in a physiologically plausible manner, which supports the validity of the phenotypic classification. This discrepancy between anatomy and molecular biology is itself informative; it suggests that ventricular morphology alone does not fully capture the biological heterogeneity of iNPH and that molecular readouts may add information regarding tissue state and clinical reversibility that is not visible on neurostructural imaging. The same argument applies to serum-based inflammatory markers, where significant CRP-IL-6^SERUM^ and LEU^SERUM^-CRP relationships in blood simply reaffirm that the cohort behaved physiologically with respect to systemic inflammatory coupling. However, PGRN^SERUM^ remained uninformative. In practical terms, this weakens the case for PGRN^SERUM^ as a stand-alone clinical biomarker in iNPH, at least in a carefully selected cohort without major systemic confounders. One could argue that this is disappointing from the standpoint of clinical convenience, since a blood-based biomarker would obviously be easier to implement than a CSF assay. Yet the negative finding is valuable, because it narrows the field and directs attention toward the compartment that appears biologically relevant. It also indirectly strengthens the specificity of the CSF signal, where the prognostic relationship was not merely driven by generalized inflammation, but by a CNS-enriched biomarker whose behavior diverged from peripheral inflammatory readouts. Another noteworthy aspect of the present results is that PGRN^CSF^ did not emerge in isolation but rather within a wide pattern suggestive of chronic intrathecal stress. Nominal associations involving lactate, total protein, and ventricular morphology and metrics may indicate that metabolic strain, BBB dysfunction, and impaired CSF turnover participate in the same disease architecture, even though the current sample size prevented these signals from reaching robust corrected significance. iNPH has been linked to altered pulsatility, reduced clearance of metabolites, periventricular ischemic susceptibility, and ependymal dysfunction, where all of these could contribute to a microenvironment in which immunometabolic compensation becomes chronically engaged [40,41]. In such a scenario, PGRN would be well positioned as a marker not merely of inflammation in a narrow cytokine sense, but of a broader neuroimmunological response to impaired CNS homeostasis. Clinically, the most defensible interpretation is therefore not that PGRN^CSF^ is a disease-specific marker of iNPH, but that it may represent a biologically informed adjunct for phenotyping probable iNPH. In this study, higher PGRN^CSF^ was associated with a less favorable short-term CSF drainage response. That finding, if validated, could be useful in several clinical scenarios: patients with a suggestive neuroradiological pattern but borderline Tap-test improvement, individuals with prolonged symptom duration, and cases in which clinicians suspect that age-related neurodegenerative burden is contributing to the phenotype. In such contexts, PGRN^CSF^ might help identify a subgroup in whom the syndrome is biologically “less reversible,” even if it remains surgically treatable. A negative association with Tap-test responsiveness should not be equated with certain shunt non-response; rather, it suggests that the immediate dynamic component of the syndrome may be attenuated in patients with greater underlying neuroimmune and tissue-remodeling burden. Future longitudinal studies should determine whether PGRN^CSF^ predicts postoperative trajectories, delayed improvement patterns, or differential response across gait, cognition, and urinary domains. Our data also speak to the broader conceptual question of whether iNPH should be understood as a singular disease entity or as a final common clinical syndrome arising from partially distinct biological routes. The heterogeneity of published biomarker findings, together with the frequent coexistence of neurodegenerative pathology in older patients, has long complicated this issue [42]. PGRN may be one component of that hidden layer of heterogeneity where patients with relatively low PGRN^CSF^ may represent a phenotype in which hydrodynamic disturbance remains more functionally dominant and potentially more reversible; those with higher PGRN^CSF^ may harbor a phenotype in which chronic neuroimmune activation, glial response, lysosomal stress, and subcortical tissue injury play a greater and more complicated role. This hypothesis is attractive because it links molecular biology to a clinically intuitive distinction that neurosurgeons and neurologists already recognize empirically: some patients appear “mechanically/shunt” responsive, whereas others present a more fixed and mixed phenotype despite suggestive imaging.

## 5. Limitations

Several limitations must be acknowledged. First, the cohort size was small (*n* = 13), which limits statistical power, inflates the uncertainty of effect estimates, and increases susceptibility to both type I and type II errors. This limitation is particularly relevant for the regression model, because complete separation and the high apparent accuracy may partly reflect overfitting despite the use of L2-penalisation, leave-one-out cross-validation, and bootstrap confidence intervals. Therefore, all predictive results should be interpreted as hypothesis-generating and require external validation. Second, the study did not include a healthy, neurodegenerative, or vascular control group; consequently, the present results cannot establish disease specificity of PGRN alterations for iNPH. The study was designed as an intrasyndromic phenotyping analysis within a stringently selected iNPH cohort rather than as a diagnostic case–control study. Thus, the principal inference is not that PGRN is specific for iNPH, but that CSF and serum PGRN behave differently and that PGRN^CSF^ tracks clinically relevant variability within probable iNPH. Third, although the exclusion criteria were deliberately strict to reduce known biological confounders of PGRN, geriatric polypharmacy remains an important potential confounder in real-world iNPH populations. Many older patients receive chronic cardiovascular, anti-inflammatory, metabolic, psychotropic, or other drugs that may influence systemic inflammation, lysosomal/autophagy pathways, microglial activation, platelet/leukocyte biology, or tissue-repair signaling. The present sample size was too small to model medication classes separately; therefore, future studies should collect medication burden in a structured manner and evaluate its relationship with both serum and CSF levels of PGRN. Fourth, the present study measured PGRN by ELISA only and did not include an independent lysosomal marker in CSF or molecular confirmation by GRN mRNA expression/qPCR. Additional lysosomal or endolysosomal markers, such as selected lysosomal enzymes or proteins reflecting lysosomal/autophagy activity, would strengthen the mechanistic interpretation that elevated PGRN^CSF^ reflects intrathecal lysosomal stress. However, the available material and study protocol did not allow valid post hoc testing of additional CSF lysosomal markers or real-time PCR. We therefore frame the lysosomal interpretation as a biologically plausible hypothesis rather than as direct mechanistic proof. Fifth, Tap-test response was used as the clinically integrated endpoint, whereas long-term postoperative outcome after shunting was not available for formal validation. Because Tap-test responsiveness is an informative but imperfect surrogate of shunt benefit, future longitudinal studies should determine whether PGRN^CSF^ predicts durable postoperative improvement and whether its prognostic value differs across gait, cognitive, and urinary domains.

## 6. Conclusions

In conclusion, the present study supports a model in which PGRN^CSF^ is not a passive bystander marker but a candidate indicator of the intrathecal neuroimmunological state in iNPH. Its dissociation from PGRN^SERUM^, positive coupling with IL-6^CSF^ and LEU^CSF^, and inverse relationship with short-term Tap-test responsiveness together suggest that elevated PGRN^CSF^ may identify a biologically more advanced or less reversible phenotype of the disorder. These findings align with the contemporary view of iNPH as a multifactorial clinicobiological syndrome in which disturbed CSF dynamics coexist with ependymal dysfunction, glial activation, impaired clearance pathways, and age-related neural vulnerability. If replicated in larger longitudinal cohorts, PGRN^CSF^ may prove useful not as a solitary diagnostic marker, but as part of a clinically interpretable biomarker framework for biological stratification and prognostic refinement in iNPH.

## Figures and Tables

**Table 1:** Descriptive statistics for all study variables organized by data group. Values are presented as mean ± standard deviation (SD) and median [Q1–Q3] for continuous variables; counts for categorical variables. SW *p*: Shapiro–Wilk normality test *p*-value for continuous variables; * *p* < 0.05 (non-normal distribution); ^†^ borderline: 0.05 ≤ *p* < 0.10.

Variable	*n*	Mean ± SD	Median [Q1–Q3]	Range	SW *p*
Anthropometric and demographic data					
Age [years]	13	76.6 ± 6.1	77.9 [72.3–82.8]	64.5–83.6	0.237
Sex [male ♂/female ♀]	8/5 months	—	—	—	—
BMI [kg/m^2^]	13	28.3 ± 4.1	28.4 [24.0–31.6]	22.3–34.6	0.466
Clinical parameters					
Elements of Hakim–Adams triad [0–3]	13	2.2 ± 0.7	2.0 [2.0–3.0]	1–3 months	0.009 *
Kiefer score (KS) [0–24]	13	6.0 ± 2.2	6.0 [5.0–7.0]	3–11 months	0.336
MMSE [0–30]	13	21.9 ± 4.4	21.0 [20.0–24.0]	15–30	0.954
WHOQOL-BREF [0–100]	13	85.3 ± 8.6	87.0 [80.8–90.8]	68–98	0.882
Duration of symptoms (SYMD) [months]	13	14.9 ± 8.5	12.0 [12.0–18.0]	6–36	0.015 *
Decision regarding VP shunt implantation [yes/no]	9/4 months	—	—	—	—
Tap-test improvement parameters					
Walking time (TTWTi) [ratio]	13	0.022 ± 0.058	0.023 [−0.012–0.035]	−0.094–0.140	0.904
Walking steps (TTWSi) [ratio]	13	0.007 ± 0.033	0.012 [−0.010–0.026]	−0.083–0.050	0.048 *
Turning time (TTTTi) [ratio]	13	0.061 ± 0.070	0.040 [0.024–0.088]	−0.058–0.175	0.546
Turning steps (TTTSi) [ratio]	13	0.029 ± 0.057	0.033 [−0.020–0.071]	−0.063–0.119	0.748
Neuroradiological parameters					
Evans index [ratio]	13	0.367 ± 0.050	0.374 [0.322–0.402]	0.288–0.443	0.633
ALVI index [ratio]	13	0.581 ± 0.063	0.589 [0.531–0.618]	0.476–0.715	0.810
Third ventricle width [mm]	13	12.9 ± 2.1	12.2 [10.9–14.6]	10.3–16.6	0.332
Callosal angle [°]	13	112.8 ± 22.2	110.8 [103.8–130.7]	69.1–146.5	0.904
Temporal horn width [mm]	13	9.8 ± 2.3	9.9 [7.8–11.6]	6.3–13.7	0.753
Blood and serum markers					
LEU (LEU^BLOOD^) [×10^9^/L]	13	6.6 ± 1.8	5.9 [5.6–6.9]	4.9–10.9	0.014 *
RBC (RBC^BLOOD^) [×10^12^/L]	13	4.5 ± 0.4	4.6 [4.3–4.7]	3.9–5.5	0.587
HGB [mmol/L]	13	8.5 ± 0.7	8.6 [8.3–8.9]	7.4–10.0	0.559
PLT [×10^9^/L]	13	234.6 ± 74.7	231.0 [187.0–247.0]	142–444	0.015 *
CRP [mg/L]	13	2.7 ± 3.8	1.1 [0.6–2.9]	0.6–14.0	<0.001
IL-6 (IL-6^SERUM^) [pg/mL]	13	4.1 ± 3.0	2.5 [1.6–5.9]	1.5–11.3	0.019 *
PGRN (PGRN^SERUM^) [ng/mL]	13	100.9 ± 10.2	99.7 [97.7–104.4]	81.7–115.6	0.434
Cerebrospinal fluid markers					
LEU (LEU^CSF^) [cells/µL]	13	3.2 ± 7.2	1.0 [1.0–1.0]	1.0–27.0	<0.001
RBC (RBC^CSF^) [cells/µL]	13	20.6 ± 35.3	5.0 [1.0–28.0]	0–128	<0.001
Total protein [mg/L]	13	436.4 ± 123.3	467.0 [321.0–522.0]	255–597	0.272
Glucose [mmol/L]	13	4.1 ± 1.4	3.5 [3.2–3.9]	3.1–7.3	<0.001
IL-6 (IL-6^CSF^) [pg/mL]	13	5.5 ± 5.6	4.0 [3.0–5.0]	2.0–22.0	<0.001
Lactate [mmol/L]	13	2.0 ± 0.4	1.9 [1.8–2.0]	1.6–3.1	0.002 *
PGRN (PGRN^CSF^) [ng/mL]	13	11.6 ± 4.2	10.6 [9.3–12.9]	6.2–22.9	0.051 ^†^

**Table 2:** Penalized logistic regression coefficients (Coef), penalized odds ratios (OR), and 95% bootstrap confidence intervals (CI)—2000 resamples, percentile method. Coefficients are shrunken L2-penalized estimates. SYMD symptom duration, KS Kiefer score.

Predictor	β	Coef	95% CI (Coef)	Penalized OR	95% CI (OR)
PGRN^CSF^	β_1_	−0.834	[−1.038, −0.361]	0.434	[0.354, 0.697]
PGRN^SERUM^	β_2_	0.032	[−0.038, 0.104]	1.033	[0.963, 1.110]
SYMD	β_3_	0.130	[−0.095, 0.507]	1.139	[0.909, 1.660]
KS	β_4_	0.632	[−0.060, 0.820]	1.881	[0.942, 2.271]
Intercept	β_0_	−0.001	—	—	—

**Table 3:** Leave-one-out cross-validation (LOOCV) performance of ridge-logistic regression models predicting the binary Tap-test response class derived from TTRV (0 = little/no response; 1 = significant response). C = 0.5 was used as the selected ridge regularization strength. Sensitivity refers to correct classification of class 1 and specificity to correct classification of class 0.

Metric	Full Model (with PGRN^CSF^)	Reduced Model (Without PGRN^CSF^)	Δ (Full − Reduced)
Accuracy	0.923 (12/13)	0.538	+0.385
AUC	0.881	0.548	+0.333
Sensitivity	1.000	0.571	+0.429
Specificity	0.833	0.500	+0.333

## Data Availability

The datasets used and/or analyzed during the current study are available from the corresponding author on reasonable request.
